# Long-Term Disease Dynamics for a Specialized Parasite of Ant Societies: A Field Study

**DOI:** 10.1371/journal.pone.0103516

**Published:** 2014-08-18

**Authors:** Raquel G. Loreto, Simon L. Elliot, Mayara L. R. Freitas, Thairine M. Pereira, David P. Hughes

**Affiliations:** 1 Center for Infectious Disease Dynamics, Department of Entomology, Pennsylvania State University, University Park, Pennsylvania, United States of America; 2 Department of Entomology, Federal University of Viçosa, Viçosa, Minas Gerais, Brazil; 3 Department of Biology, Pennsylvania State University, University Park, Pennsylvania, United States of America; Universidade de São paulo, Brazil

## Abstract

Many studies have investigated how social insects behave when a parasite is introduced into their colonies. These studies have been conducted in the laboratory, and we still have a limited understanding of the dynamics of ant-parasite interactions under natural conditions. Here we consider a specialized parasite of ant societies (*Ophiocordyceps camponoti-rufipedis* infecting *Camponotus rufipes*) within a rainforest. We first established that the parasite is unable to develop to transmission stage when introduced within the host nest. Secondly, we surveyed all colonies in the studied area and recorded 100% prevalence at the colony level (all colonies were infected). Finally, we conducted a long-term detailed census of parasite pressure, by mapping the position of infected dead ants and foraging trails (future hosts) in the immediate vicinity of the colonies over 20 months. We report new dead infected ants for all the months we conducted the census – at an average of 14.5 cadavers/month/colony. Based on the low infection rate, the absence of colony collapse or complete recovery of the colonies, we suggest that this parasite represents a chronic infection in the ant societies. We also proposed a “terminal host model of transmission” that links the age-related polyethism to the persistence of a parasitic infection.

## Introduction

High density living in human settlements, or among the animals and plants we raise for food, can result in both major epidemics and the emergence of novel parasites [1]. Dense societies also occur in natural systems and the prime example are the social insects. Their colonies can contain thousands and sometimes millions of highly related individuals [2], which might suggest repeated epidemics. However, insect societies have in fact achieved both ecological dominance and long evolutionary stability. For instance, ants have become dominant in terrestrial biomes accounting for over 50% of the biomass despite making up less than 2% of all insect species [3]. This success indicates that their societies have evolved ways to reduce disease pressure. A prominent defense mechanism of insect societies is the social immunity, which is mounted collective actions to prevent and control diseases spread in benefit of themselves and the others [4]. Therefore, important lessons for limiting disease spread might be gained from examining societies that have evolved by the process of natural selection over long periods of time.

Many studies have investigated how ants, termites, bees and wasps behave when a parasite is introduced into their colonies. Especially for ants, several studies have demonstrated that collective behavior and positive interactions between nestmates increases the survivorship of infected individuals, and decreases the probability of new infections and disease outbreak [4]–[12]. Interestingly, no studies have yet shown social behavior to have no effect or to accelerate disease transmission. A key feature of these studies is that they have been conducted in the laboratory, so our understanding of the dynamics of ant-parasite interactions under natural conditions remains very limited.

In nature, insect societies are host to a high diversity of macro and microparasites [6]. While some are generalists, able to infect hosts from different taxa, others are specialists, with a narrow range of hosts. The challenge for both generalists and specialists has been to evolve strategies to overcome and/or avoid host defenses. To fully understand the evolution of ant societies within the context of infectious diseases we need to study such systems in nature. Here we complement previous laboratory studies on social immunity with studies of parasites of ants within a rainforest. Focusing on a specialized fungal parasite, we first established the parasite prevalence in population level within an Atlantic rainforest fragment. Secondly, we conducted a long-term detailed census of parasite pressure surrounding four colonies by mapping the precise locations of fungus-killed ants and the position of foraging trails. Our study of diseases in an ant society within a complex natural environment will help us understand how parasites can persist in the host population despite the host’s effective disease surveillance.

## Materials and Methods

### Study area and host and parasite species

Fieldwork was carried out at the Research Station of Mata do Paraíso, Universidade Federal de Viçosa, Minas Gerais, Southeast Brazil (20°48′08 S 42°51′31 W). The carpenter ant *Camponotus rufipes* is very abundant in this habitat. The ants forage on trails, being active at night with activity peaking in the early evening [13]. The trails are built mainly on twigs and branches lying on the forest floor so the ants use the 3D space of the forest, not walking only on the floor. Ant trails are permanent with the same trail being used for weeks [13].

The newly described parasitic fungus *Ophiocordyceps camponoti-rufipedis,* previously known as *O. unilateralis* [14], is a parasite that has *C. rufipes* as its host, and it is also abundant in the study area [13]. It is a specialized parasite of ants that must kill the host to complete its life cycle. Before killing the infected ant, the fungal parasite manipulates the behavior of the host, leading the host to climb the vegetation and bite the veins or margins of leaves in rainforests [15]–[18]. The manipulation is followed by the death of the ant, which remains attached to the leaf postmortem. Dying attached to a leaf is not normal behavior and only happens because of the manipulation by the fungus [15], [17] Thus, all the ants discovered in this condition (dead, biting and attached to leaves) had been infected and manipulated by the parasite. The dead ant attached to the leaf serves as a platform for fungal growth [17]. The fungus *O. unilateralis sensu lato* has a stereotyped development that first requires the transition of the spores within the host body to hyphae, which coordinate to form a stalk (stroma), followed by the formation of the ascoma in which transmissible spores develop. The ascoma is a large round structure on one side of the stroma (hence the epithet, *unilateralis*). Inside the ascoma the infectious spores, called ascopsores, develop and are forcibly discharged onto the forest floor underneath the cadaver. The crucial step before spore release is the coordinated growth of the hyphae to form the stroma that happens between 7–10 days after host death [15], [18]. Thus, the fungus *O. camponoti-rufipedis* kills its ant host as a developmental necessity and post-mortem there is a stereotyped sequence of development before transmission can occur.

### Disease within the nest

Experimentally it has been shown that the preliminary and vital first stage of development (stromatal growth) cannot happen if the ant cadaver is placed either on the forest floor or in the dry upper canopy of the rainforest [15]. This implies that initial development is very sensitive to the microclimate. Thus, the manipulation where infected ants bite leaf tissue in the understory vegetation before dying appears adaptive for the parasite [15]. While informative, these earlier experiments left open the question whether the parasite could develop inside the nest of the host. To determine whether the fungus is able to complete the first necessary step towards transmission (i.e. grow the stroma) inside an ant mound, we collected a whole nest of *C. rufipes* (including nest material, ants and brood) and a recently abandoned nest (only the nest material, no ants or brood). It was possible to collect the nest because these ants build a characteristic semi-spherical mound composed off small dry leaves and twigs in the base of trees, allowing us to identify the species by the nest [19]. Abandoned nests are easily identified and collected.

Both nests (with and without ants) were directly placed in buckets (volume = 8 L), maintaining the original characteristics of the nest, and kept under a natural day/night light and temperature regime. We collected 28 *C. rufipes* ants freshly killed by *O. camponoti-rufipedis* from the same forest fragment where we collected the nests. In each cadaver, the fungus was at the early pre-stroma stage of growth. Hyphae had grown through the intersegmental membranes of the ants and were beginning to form what would be the base of the stroma. Since the stroma precedes ascoma development and spore dispersal it is a crucial stage for the transmission. Failure to grow the stroma results in zero fitness for the parasite. Although the samples and nests used for the experiment were collected in the same fragment, it was not possible to know from which colony the dead ants originated. However, since our aim here is to investigate parasite development, that occurs outside the colony, we assume that the colony of origin should not affect the parasite after it has killed the host.

We took pictures of the initial conditions of each of the 28 samples and attached them to flags (the flags facilitated recovery of the samples once the experiment concluded). The 28 ants were placed in two different treatments: (1) nest with ants, n = 14; (2) nest without ants, n = 14. In the treatment with ants, 50% sugar/water and canned tuna were provided to feed the adult ants and larvae. The same number of water tubes was added to the bucket without live ants to ensure the abiotic conditions would be the same in both treatments, and the only variation between the buckets was the presence or absence of live ants. In both treatments the fungal killed ants were placed on the top of 10 cm of nest material and covered with 20 cm of the same nest material. The ant cadavers were removed 10 days later, which is the period of time in which the crucial growth of the stroma occurs. After the 10 days, photographs were taken to evaluate the development of the fungus.

### Disease surrounding the nest

Because social immunity is well known from experimental studies in the laboratory to be effective and rapidly deployed [5], [7], [8], [10]–[12], [20] we might expect some colonies within a population to be free of *O. camponoti-rufipedis*. We therefore first set out to ask how common infection by *O. camponoti-rufipedis* was at the population level. In order to identify nests of *C. rufipes*, we made 22 transects of 2,000 m^2^ each (100×20 m), covering 44,000 m^2^. The first 16 transects were initiated perpendicular to the main path of the research station, and proceeded 100 m into the forest. Along the 100 m transect we searched 10 m on both sides so that we examined 2,000 m^2^ for each transect. In order to obtain more complete coverage of the site, we delineated a new path from which we traced the other six transects, covering an extra 12,000 m^2^. The distance between the start points of each transect was 100 m and the transect direction alternated between the left and right sides of the path. Using this transect approach we found 9 nests. Another 8 previously identified nests were used in this study. For these 17 nests we examined the vegetation within a 1 m radius around each nest looking for ants killed by the fungus *O. camponoti-rufipedis.* Killed ants are prominent as they are attached by their mandibles to the underside of leaves. The locations of all the nests were recorded using a GPS and the nests that had dead ants in the adjacent vicinity were recorded.

To investigate the disease surrounding the nest in more detail and over longer periods of time, we mapped, in Euclidean space (i.e. 3-dimensions, x, y and z – see Table S1), the dead ants surrounding 4 nests over 20 months (Dec 2010–Jul 2012). Because ants are known to travel long distances from their nests [21], we limited the mapping to the area surrounding the nest. We focused our survey in this area because, independent of any changes in the trails’ directions, the ants will walk through the area surrounding the nest when leaving or returning to it. For 4 of the 17 previously discovered nests, we demarcated a study area of 200 m^3^ (10 m×10 m×2 m) (henceforth called ‘plot’) that were centered on the nest. Thus, we observed the long-term dynamics (20 months) of the fungal infection in four distinct colonies.

In order to determine the 3D position of ants killed inside the four studied plots, we used the coordinate system relative to the nest, determining the x, y and z position of each dead ant, using the left bottom corner as the origin. For example, all the four nests had the coordinates (500, 500, 0) because they were in the center of the plots (x = 500 cm, y = 500 cm) and on the forest floor (z = 0 cm). We measured the infected dead ants in 3D (x, y, z coordinates) because the ants are manipulated to die attached to the underside of leaves on plants in the understory vegetation of tropical forests [14]–[18].

Before beginning the 3D measurements, in November 2010 we tagged all dead ants in those plots by checking every single leaf inside the plots, up to 2 m from the forest floor. Across the first six consecutive months (December 2010–May 2011) we identified, tagged and mapped (x, y, z coordinates) every single newly killed ant attached to leaves within each of the four plots. None of the dead ants that we counted were removed from the plots so we did not reduce the naturally occurring parasite pressure. To capture long-term dynamics, we left the area for seven months following the May 2011 census and mapped the new cadavers in January 2012. Finally, we returned in July 2012 to check if each of the four nests had new dead ants on the immediate vicinity of the colony.

We limited our survey to the immediate area surrounding the nest because this is the zone the ants must walk through to leave and return to the colony. To better understand the path workers ant took we also measured and mapped in 3D the trails formed by the ant. This also allowed us to access spatial location of the potential new hosts, which would be on the foraging trails. The foraging trails were marked with small flags placed every 30 cm, starting at the nest and continuing until they left the plot. The coordinates of each flag inside the area were determined the same way as we did for the dead ants. The z positions, measured from the forest floor, were included because the trails pass along on branches, lianas and roots above the forest floor [13]. Combining those coordinates we were able to access the exact location of each trail in space, related to the nest. We also did not disturb the ant trails.

The trails and dead ants were mapped once a month. For each plot, one whole day was necessary to map dead ants and trails. During the daylight hours we recorded the positions of the dead ants, and at night time the trails, since the ants are nocturnal [13]. The 3D data were plotted using the Grasshopper plugin for the 3D modeling platform Rhino. Statistical analyses were conducted using R (version 2.15.2). We tested if the number of dead ants was dependent on months and ants activity (estimated by the number of trails). We used generalized mixed models to avoid temporal pseudo-replication, using the variable “Month” as repeated factor.

## Results

### Disease within the nest

Of the total of 28 *O. camponoti-rufipedis* samples (and their ant cadavers) placed inside the nests, none developed the stroma (Fig. S1, S2). For the nest containing live ants, 9 (64%) out of 14 cadavers were removed from the leaf they were attached to and it was not possible to find them (the leaves were recovered but not the cadavers, suggesting they had been broken up by the live ants). The fungus in the remaining 5 cadavers (36%) did not grow the stroma correctly. Within the 14 samples placed in the bucket containing only nest material (absence of live ants), 8 (53%) of them did not grow at all. The remaining 6 (47%) grew abnormally, having shorter, corkscrew shaped stroma, in a way that ascoma formation and subsequent spore transmission would not be possible. In summary, since the spore production occurs from the ascoma, the specialized structure that grows after the development of stroma (Fig. S1 B, D), all 28 samples of the fungus failed to develop the reproductive mature stage, which is required for transmission. Even if we do not consider the 9 cadavers that were removed from the bucket with live ants, our remaining 19 samples still allow us to conclude the fungal parasite was incapable of reaching the infective stage inside ant nests, whether ants were present or not (Fig. S2). To confirm the conditions outside the nest were suitable for the normal development of fungus, we searched for more dead infected ants (outside the plots) and recorded their developmental stage. We found 248 cadavers with fungal development ranging between initial stroma formation (minimum of 0.5 cm) and mature reproductive stage (stroma and ascoma fully developed). We are also able to exclude experimental manipulation of the cadavers as a factor as we do this routinely, without effects on fungus development.

### Disease surrounding the nest

We discovered 17 nests that were patchily distributed in the study area. All 17 nests had ant cadavers attached to leaves beside the ant colony. Thus, the prevalence of this host-specific parasite at the population level is 100%. As we searched for colonies in the entire area it is unlikely that this high prevalence is a product of subsampling within the area.

**Figure 1:**
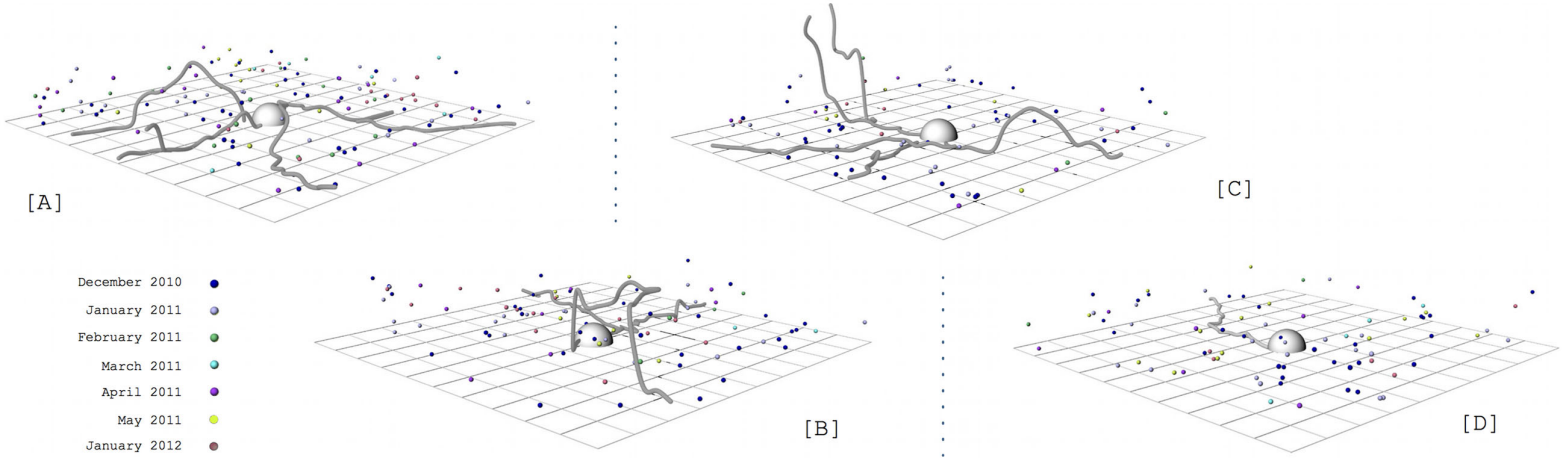
3D maps of foraging trail and monthly-infected ants surrounding ant colonies in Atlantic rainforest, Brazil. The infected ants represent accumulated dead ants in 7 months (Dec 2010–May2011 and Jan 2012). Distinct colors represent different months. The lines show trails were recorded in December 2010. (A) Nest A. (B) Nest B. (C) Nest C. (D) Nest D.

**Figure 2:**
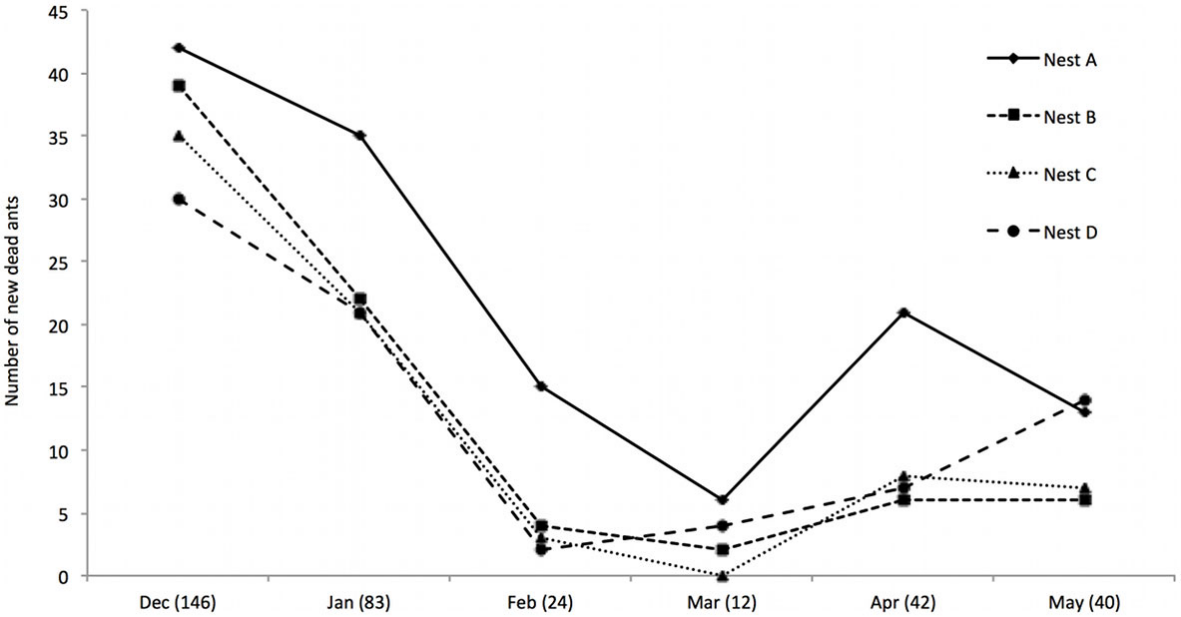
Disease dynamics surrounding four nests of *Camponotus rufipes* in an Atlantic rainforest fragment, Brazil. Different lines represent each analyzed nest (A, B, C or D). The numbers show the total of dead ants recorded for each of the six months.

In the first census we conducted, in November 2010– which preceded the monthly 3D mapping – we recorded 358 ant cadavers attached to leaves. In the following 6 months (December 2010–May 2011), we identified 347 newly dead ants, killed by *O. camponoti-rufipedis* within the areas surrounding the four colonies (Fig. 1, Movie S1, Table S1). The number of dead ants was month-dependent (Mixed-model: χ^2^_5_: 60.877; P<0.0001). December 2010 had the highest density of parasitized ants: 146 dead ants attached to leaves were found in the census for that month (Mixed-model: χ^2^_1_: 18.052; P<0.0001) (Fig. 2). The lowest occurrence of dead ants was in March 2011, when we recorded a total of 12 dead ants; but this did not differ statistically from February (24 dead ants) (Mixed-model: χ^2^_1_: 2.0164; P = 0.1556) (Fig. 2). November, December and January receive 75% of the yearly precipitation [22], which is likely an important determinant of abundance for fungal parasites.

Our second census was in January 2012, after the plots had been left untouched for 7 months. We found a total of 39 new dead ants within the 4 plots (that is, after seven 7 months, each nest had freshly killed cadavers attached to leaves). Finally, when we returned in July 2012 and established that, even after 20 months, each of those four nests had new infected manipulated ants that died in the surrounding area of the nest, demonstrating the long-term persistence of the parasite surrounding these colonies.

Over the 20-month period we quantified the infected dead ants in eight months (i.e. months 1–6, 7, and 20) surrounding four colonies. Only once and for only one colony we did not find new records of *O. camponoti-rufipedis* surrounding the host colony (Nest C) (Fig. 2). This was the month that the density of new cadavers was lowest for all colonies (March 2011) (Fig. 2). However, in the following month (April 2011), we did find newly manipulated killed ants outside Nest C, demonstrating that the colony was not disease free. The resurgence of dead ants around the colony C in April, after a month without records, could be explained by: (i) cadavers from previous months were still sporulating, as some cadavers remained across months; (ii) the infection covers an area beyond the surrounding vicinity of the nest, as we found cadavers outside the plots.

Because we measured and mapped the position and abundance of ant trails, we also investigated the role of host activity on the disease dynamics. We did not specifically count the number of ants in each trail, but after 8 months of nocturnal observations of natural foraging trails, we established that the flow of ants in each trail of a particular nest was roughly the same. It is important to note that the flow of ants among nests was variable [13], but not within trails of the same nest. Thus, we assumed the number of trails for each month which, based upon our extensive observations, is a reasonable representation of the healthy ants susceptible to new infections. The number of trails was related with the number of dead ants of each month. If the primary limitation for new infections were the number of suitable hosts in the system, we would expect to find more dead ants when the ants were more active (activity was measured by number of trails). However, the number of infected ants attached to leaves surrounding the colony was not correlated with the number of susceptible hosts (Mixed-model: X^2^_1_: 2.1078; P = 0.1466).

## Discussion

Many laboratory studies have demonstrated that social immunity is an important feature of insect societies, notably ants [5], [7], [8], [10]–[12], [20]. For the first time, we have found evidence of the occurrence of social immunity in ant societies under field conditions. Our results support the theory of social immunity operating inside the nest of social insects, as we have shown that the *Camponotus rufipes* ants removed most of the *Ophiocordyceps camponoti-rufipedis* parasitized cadavers placed within the nest. Although we did not know the colony of origin of the dead infected, it should not affect our results because necrophoreses – removal of corpses – is a common behaviour within diverse species of ants [23]–[25], including *Camponotus* species [26]. Previous studies on disease in ant societies interpreted necrophoresis [25], [27], [28] and social isolation [10], [29] as a class of behavioral immunity that prevents diseases spreading among nestmates [10], [27]–[29]. However, and importantly, we also found that this specialized fungal parasite, when placed inside a nest without ants, cannot grow to the stage suitable for transmission (that is the stroma and ascoma). Therefore, simply being within the nest reduces the fitness of the specialized parasitic fungus *O. camponoti-rufipedis* to zero, whether the nest is inhabited by ants or not. The growth inside the nest may be constrained, primarily, by the physical limitations. To successfully develop, the stroma must grow roughly one body length of the ant and this growth is perpendicular to the posterior-anterior axis of the ant to reach the successful development of the fungus. Secondly, social insects are known to control the nest climate [30], and *O. unilateralis s.l.* has been shown to be very sensitive to microclimate [15]. Lastly, the destruction of the cadaver by healthy ants is also a potential limitation for the fungus to develop inside the nest, reinforcing the hypothesis that species of fungi in the complex *O. unilateralis s.l* cannot grow inside ant colonies, because of the behavior of the ants, physical limitation for stromatal/ascomatal growth or microclimate conditions.

It may be that the removal of corpses and, more importantly, dying in social isolation (outside the nest) actually increases the opportunity for the parasite to complete its development and be transmitted to the next host. From the perspective of the colony, the ability of nestmates to destroy cadavers before the fungus can become infectious - which probably happened to the cadavers that we could not recover - means that remaining inside the nest might better serve the colony compared to ants dying in social isolation outside the nest where fungal growth can occur. The same may apply for generalist pathogens, such as *Metarhizium* (used in the majority of studies on social immunity), that have a broad range of hosts [31]. The fungus *Metarhizium* can be transmitted from an infected ant to other insect groups outside the nest when the ants display the behavior of isolation before death [10] or when the colony removes corpses [23]–[25]. The development of *Metarhizium* would be hampered if the corpse were destroyed inside the nest before sporulation (*Metarhizium* also requires host death preceding the sporulation). In laboratory experiments, it is common to collect the post-mortem ants to confirm the cause of death, so the development of generalist parasites within the nest remains to be studied.

Not all parasites of ants use the same strategy of manipulating the host as the species of *Ophiocordyceps* that we studied. To place our results within the wider context of parasites evolved to infect ant societies we examined the mode of transmission for other specialized parasites of ants (Table S2). It was striking that, as with *Ophiocordyceps,* the majority of parasites of ants require the infected ant to leave the nest to continue the life cycle. Social isolation mediated by parasites may be a widespread strategy in parasites that attack ant societies. These parasites only can only be within the nest when they are invisible to the nestmates, that is, within the infected ant body. The life stage that requires them to either exit from or protrude from the host body occurs outside the nest, where social immunity does not act.

Although social immunity is present in insect societies such as the ants studied here, and does function to prevent disease transmission within the nest, our full appreciation of it may not be wholly realized because to date we have been biased by studies that have focused solely on ant behavior towards diseases inside the nest. All the 17 colonies we found in the area were infected by the specialized parasite. More important, the infections by *O. camponoti-rufipedis* were persistent over time, with new ants being killed every month. At first sight, combining the absolute prevalence (i.e. 100%) among colonies and the persistence over time, the parasite seems to be a serious threat for the colonies. However, we never observed the death of any of the colonies, suggesting the parasite exerts low pressure over them. Accordingly, the trails emerging from the colonies did not seem to respond to the natural levels of infection: over 20 months of fieldwork, the ants did not cease their activity. We also know, from a study conducted in the same area, that *C. rufipes* maintains the same trail pathways for long periods [13]. Such trail fidelity might explain the absence of a correlation between colony activity level and number of new infected ants. Considering that the parasitic fungus is very sensitive to microclimate [15] and because we found more dead ants during the months with higher precipitation, it is plausible that the abiotic conditions play more decisive role in an environment where suitable hosts will always be available (i.e. ants never cease the forager trails).

Although we did not observe colony collapse, we also did not observe any colonies clearing the infection. For each month we conducted the census, we found new dead ants surrounding the colonies (average of 14.5 cadavers/month/colony). This implies that the parasite does constantly consume workers, even if this is a relatively small number of workers. It is also important to note that we limited the census to the immediate vicinity of the nest although we know there were dead ants beyond the plots we studied. This means that our census might be an underrepresentation of the number of workers ants that are lost to the colony because of this parasite. Thus, the continuous baseline fitness loss caused by the parasite functions as a long lasting condition for the colony, characterizable as a “chronic disease” that, as in humans, can be controlled but not cured.

### The terminal host model of transmission

Social immunity is effective and prevents disease transmission within the nest. From a host-centric view this would appear to provide an advantage to the host within a supposed arms race between the two parties. However, we offer an additional viewpoint. The fungus *O. camponotini-rufipedis,* infects susceptible hosts (foraging ant workers) by means of large curved spores, which fall directly down from the cadavers attached to leaves, that will be picked up by a new host [14]. Foraging for food is vital for the colony and workers must perform this task. Ant colonies are predicted to work as a “conveyor belt model” [6], following an age-related polyethism where they generally start with internal tasks and gradually transit to tasks outside the nest as they age [24]. Typically, these tasks outside the nest (e.g. foraging) are very risky, and are carried out by older workers, which are going to die sooner [32], [33]. In this scenario, where the older ants collect the food supplies required by the colony, there is a constant turnover of new susceptible ants on trails. We expect that the proportion of infected ants within a colony is low, since not all workers forage. As we have shown, the foragers are constantly being killed by the parasite, and new workers will take over the risky tasks that are done outside the nest, providing a continual stream of new hosts for the parasite that sits outside the colony. Probably over the longer term such a strategy has impact son the host demography and social interactions, although evidence remains lacking on this important question.

Infecting a specific group within a population or group of cells within a body is a widespread strategy in antagonistic interactions. For instance, many predators attack weak prey, which include old, sick, and young individuals. These are easier to capture as they occupy peripheral positions on the outside of the herd or simply lag behind in chases and, because of their weak status, are undefended. For within-body host-parasite interactions, the papillomavirus uses the strategy of a high reproductive rate in terminal cells, which is considered advantageous because there is no immune surveillance in such cells [34]. This virus, which is transmitted by contact, forms warts on the most external surface of the host body - skin, enabling the transmission to a new potential host [34]. We hypothesize that the specialized parasite *O. camponotini-rufipedis* (and other *Ophiocordyceps* species infecting ants) specifically infects older individuals from ant societies and causes them to die outside the nest. The advantage is that the parasite does not need to evolve mechanisms to overcome the effective social immunity that occurs inside the nest, and at the same time, it ensures a constant supply of susceptible hosts, resulting in a “chronic infection”.

An option for the host would be extending the social immunity to the outside nest environment. There are anecdotal observations of ants removing fungus-manipulated and killed cadavers from the environment. In the Amazon rainforest, the turtle ant, *Cephalotes atratus*, which is arboreal, removes the cadavers from the bark of trees [35]. The wood ant, *Formica rufa*, which inhabits grasslands, removes the cadavers of infected ants manipulated to die on the immediate vicinity of the ant nest and trails [36]. It would be of great interest to test how far out from the colony social immunity can extend. In the 20 months of fieldwork we did not see any ants destroying or removing the cadavers attached to leaves surrounding the nest, leading us to suspect that they do not display the same defensive behavior around the nest as they do inside. It is likely difficult and costly for ants to control the outside environment, where *O. camponoti-rufipedis* is found. Although social immunity was not detected outside the nest in this case, it might be possible for adaptive changes in ant behavior to reduce the disease burden. The species of ant we studied builds their foraging trails using bridges and this might function to reduce contact with the soil and establish the permanent use of the same pathway, both of which might decrease the risk of infection [13], [37]. We do know examples of how the foraging trail network of ants adaptively shifts in response to changing food abundance [38], [39] or to reduce the incidence of attack by predators [40]–[42] or competing colonies [43], [44]. There are also examples of trails shifting in presence of parasitoid females that lay eggs in workers [45]–[47]. Also, the presence of *O. unilateralis* in Thailand was suspected of causing the target ant, *Camponotus leonardi* to reduce the time spent near the forest floor [15]. Generally, ant trail behavior and its response to parasites are neglected but with our focus on within forest parasite-host dynamics we hope to encourage such work. However, because foraging ants tend to be older there may simply be little selection on the host to evolve strategies against the parasite. If this is the case then host and parasite may not be involved in an arms race (as is commonly assumed) at all. They may both be following quite stable evolutionary strategies in which the parasite is tolerated by the host.

### Conclusion

To date, our knowledge on effective collective action of ants against infections has focused on the host response to parasites introduced within the nest. Here we described the natural dynamics of a specialized parasite of ant societies within a rainforest. The disease census we conducted over 20 months revealed that the ant *C. rufipes* is constantly infected by the parasite *O. camponoti-rufipedis*. Although the social insects nests appear to represent the ideal conditions for disease spread, this parasite can only grow and transmit outside the host nest, as is indeed the case for many other specialized parasites of ants (Table S2). We suggest that the transmission outside the nest is advantageous for the parasite because it avoids the different levels of social immune responses and may still have access to new hosts, since the workers (typically the old ones) need to leave the nest to collect food. For the first time, we have linked the age related polyethism type of social organization, previously interpreted as a component of social immunity, to the persistence of a parasitic infection. Based on the lower infection rate, absence of colony collapse or complete recovery of the colonies, we suggest that this parasite represents a chronic infection in ants’ societies. There is no doubt that social immunity is an essential component of the social insects life style, but we suggest the parasites may have evolved to avoid it or exploit some of its components.

## Supporting Information

Figure S1 ***Camponotus rufipes*** **ants infected infected by** ***Ophiocordyceps camponoti-rufipedis*** **.** (A) Ant recently killed by the specialized parasite *Ophiocordyceps camponoti-rufipedis.* (B) Mature *O. camponoti-rufipedis* stage, suitable to transmission. The arrow points to the frutification body from where the spores are shot. (C) Collected ant recently killed by the fungus parasite before on the experiment. Fungal presents initial development (arrow). (D) Same sample after 10 days inside the host nest. The fungal did not developed as it normally does outside the next (arrow).
(TIFF)

Figure S2 **Data collected for the experiment** ***Disease within the nest*** **.** Before (day 0) and after (day 10) pictures for 18 cadavers recovered from the host nest after 10 days. The samples 1 to 14 were placed within a nest with live ants and samples 15 to 28 were placed within the nest material in absence of live ants (as described in our materials and methods section).
(PDF)

Table S1 **Relative position (coordinates) of the 386 mapped infected ants found on the surrounding area of the four studied colonies across 20 months of field work.** The data was collected in Atlantic rainforest, southeastern Brazil. The fungal parasite species is *Ophiocordyceps camponoti-rufipedis* that attacks the ant host *Camponotus rufipes*. The ant colonies were located in the center of the studied area and their coordinates are (x = 500, y = 500, z = 0). Months 1–6 correspond to December 2010 to May 2011. Month 14 corresponds to January 2012.
(PDF)

Table S2 **Overview of co-evolved parasites in ant societies.** Transmission can be between ants (direct) or also include another host (indirect). The final environment, where the sexual reproduction of the parasite occurs, can be in the environment surrounding the nest (Outside the nest), within the colony (Inside the nest) or final host (Vertebrate host). The effect of parasitism is often death of the infected, either directly attributable to the parasite (Direct death), or indirectly via a behavioral change that leads to the host being eaten by the final host (Predation) or jumping in water, to allow the parasite to enter water for mating (Drowning). Additional details of each group in Schmid-Hempel (1998) [6].
(DOCX)

Movie S1 **Spatiotemporal dynamics of the specialized fungal parasite attacking an ant colony across six consecutive months.** The data was collected in Atlantic rainforest, southeastern Brazil. The fungal parasite species is *Ophiocordyceps camponoti-rufipedis* that attacks the ant host *Camponotus rufipes*. The red dots represent the new ants killed by the parasite in the respective month. The grey dots represent the sum of dead ants from previous months. The red lines represent the forage trail on the ant host for each studied month.
(MP4)
