# Supplementary figures and images for: Long-Term Disease Dynamics for a Specialized Parasite of Ant Societies: A Field Study
Source: PLoS One. 2014 Aug 18;9(8):e103516. doi: 10.1371/journal.pone.0103516 (PMC4136743; doi:10.1371/journal.pone.0103516)

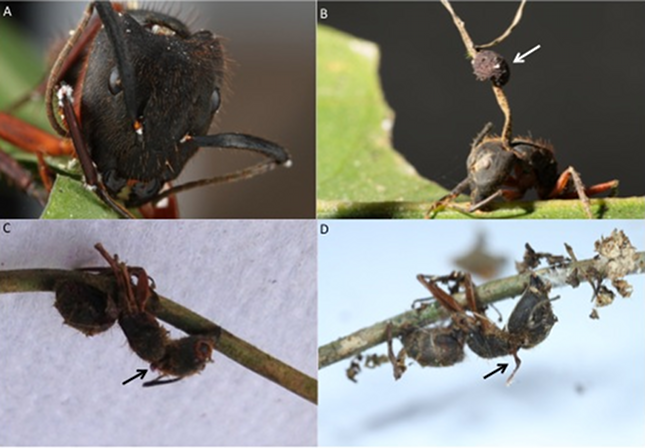

Supplement: Figure S1 — Camponotus rufipes ants infected infected by Ophiocordyceps camponoti-rufipedis . (A) Ant recently killed by the specialized parasite Ophiocordyceps camponoti-rufipedis. (B) Mature O. camponoti-rufipedis stage, suitable to transmission. The arrow points to the frutification body from where the spores are shot. (C) Collected ant recently killed by the fungus parasite before on the experiment. Fungal presents initial development (arrow). (D) Same sample after 10 days inside the host nest. The fungal did not developed as it normally does outside the next (arrow). (TIFF) [file pone.0103516.s001.tiff]
