# Supplementary material for: Long-Term Disease Dynamics for a Specialized Parasite of Ant Societies: A Field Study
Source: PLoS One. 2014 Aug 18;9(8):e103516. doi: 10.1371/journal.pone.0103516 (PMC4136743; doi:10.1371/journal.pone.0103516)
Supplement: Figure S2 — Data collected for the experiment Disease within the nest . Before (day 0) and after (day 10) pictures for 18 cadavers recovered from the host nest after 10 days. The samples 1 to 14 were placed within a nest with live ants and samples 15 to 28 were placed within the nest material in absence of live ants (as described in our materials and methods section). (PDF) [file pone.0103516.s002.pdf]

Inside nest experiment data

Smaples 1-14: nest with live ants. Samples 15-28: nest without live ants (only nest material)

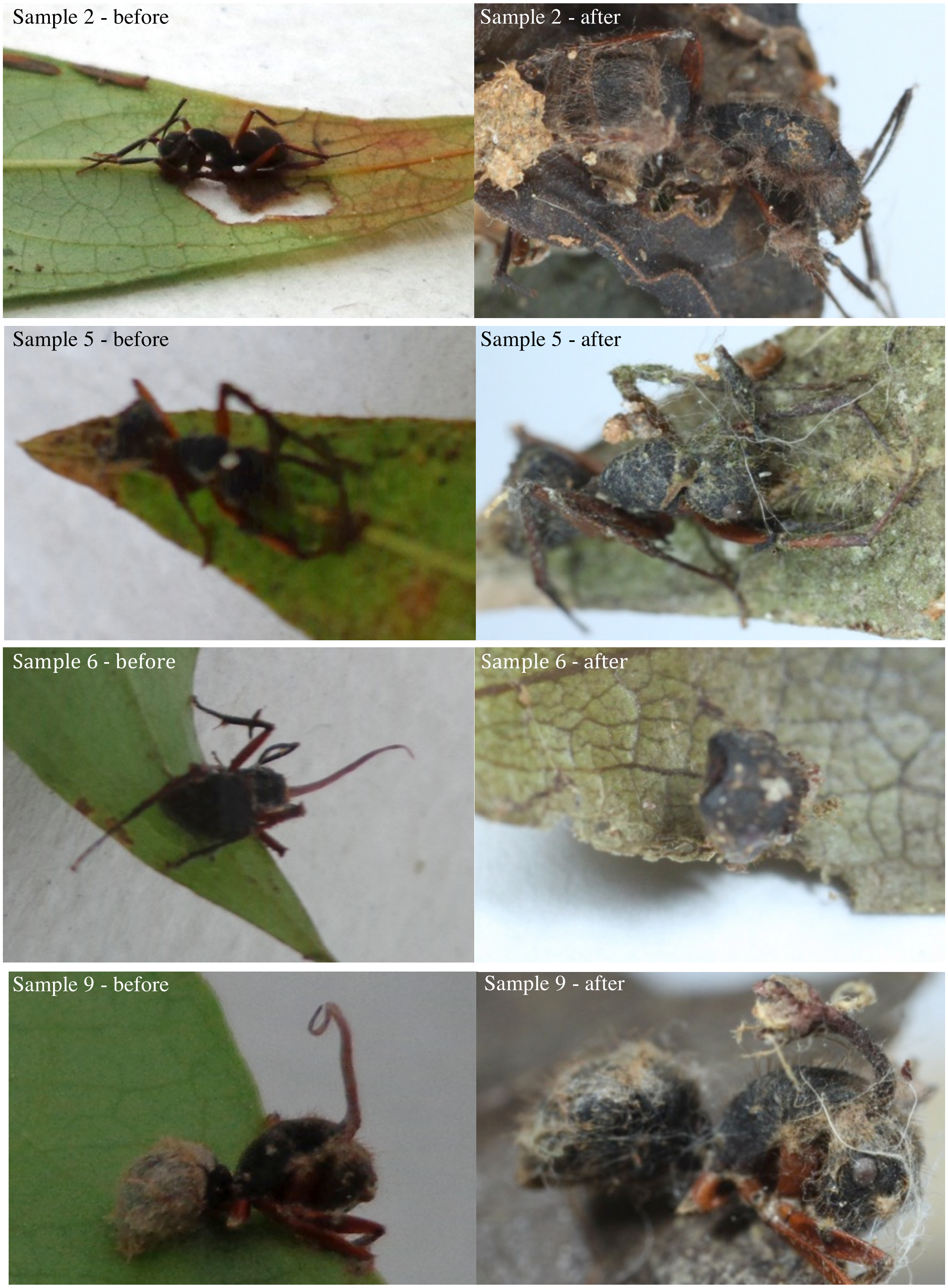

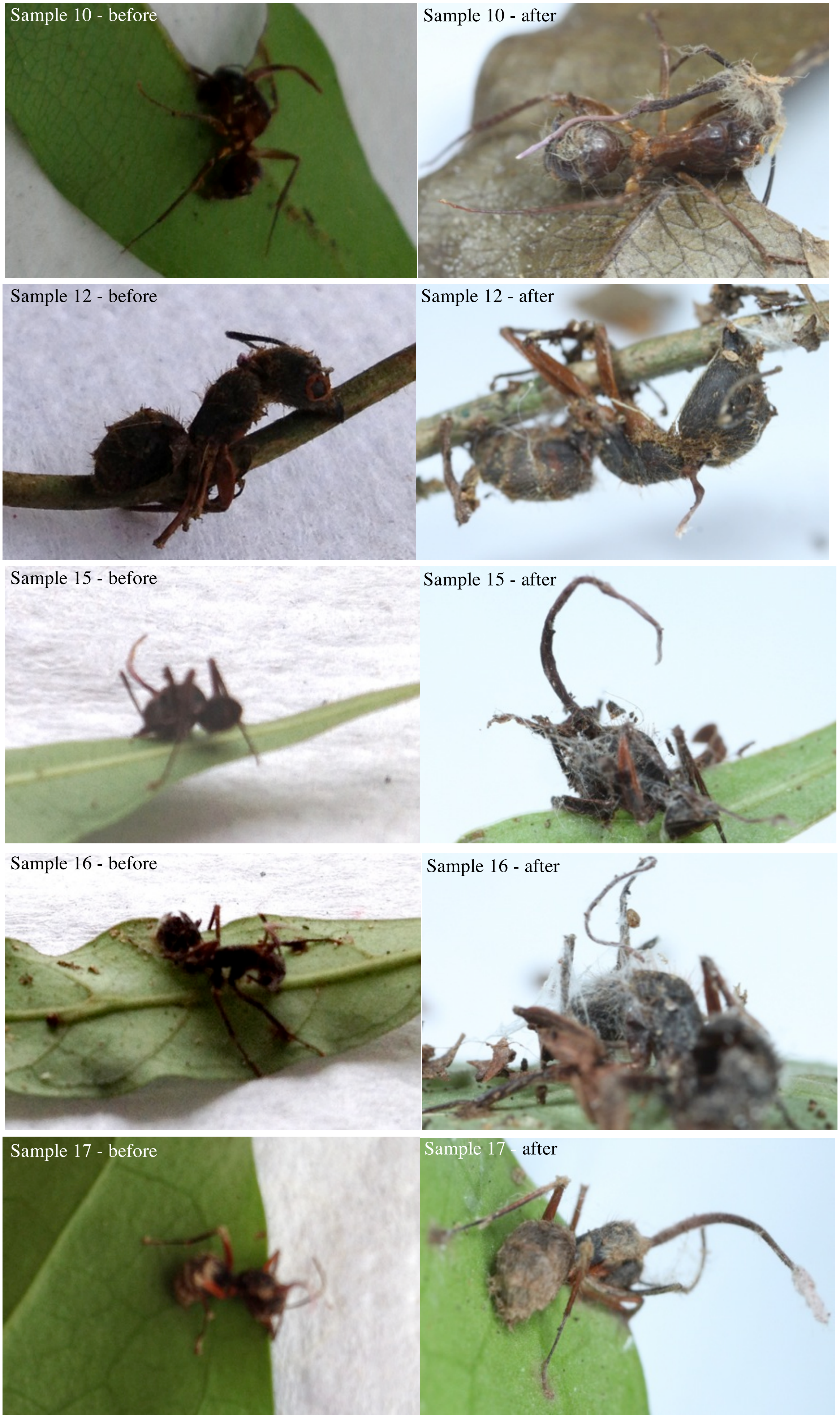
Sample 10 - before
Sample 10 - after
Sample 12 - before
Sample 12 - after
Sample 15 - before
Sample 15 - after
Sample 16 - before
Sample 16 - after
Sample 17 - before
Sample 17 - after

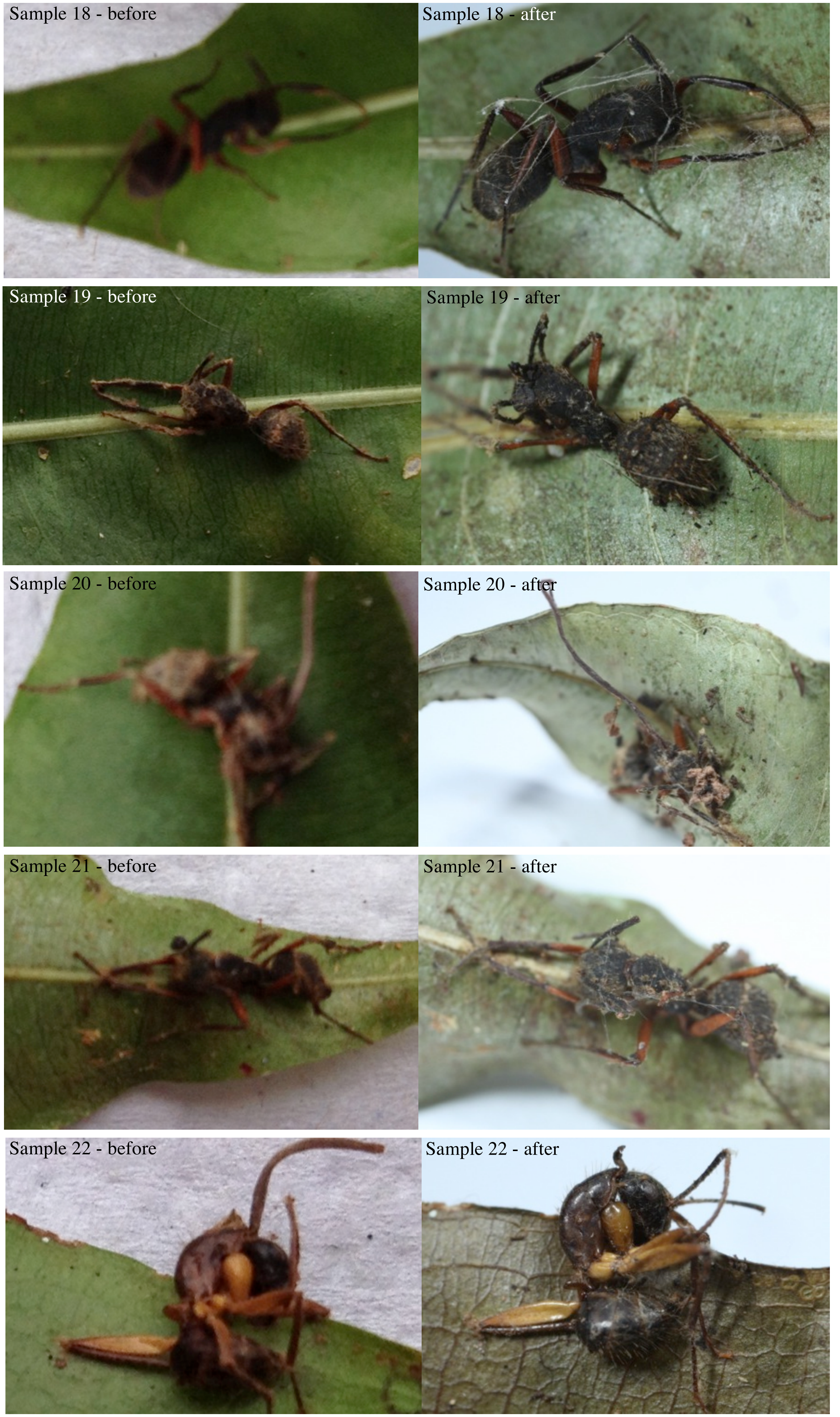
Sample 18 - before
Sample 18 - after
Sample 19 - before
Sample 19 - after
Sample 20 - before
Sample 20 - after
Sample 21 - before
Sample 21 - after
Sample 22 - before
Sample 22 - after

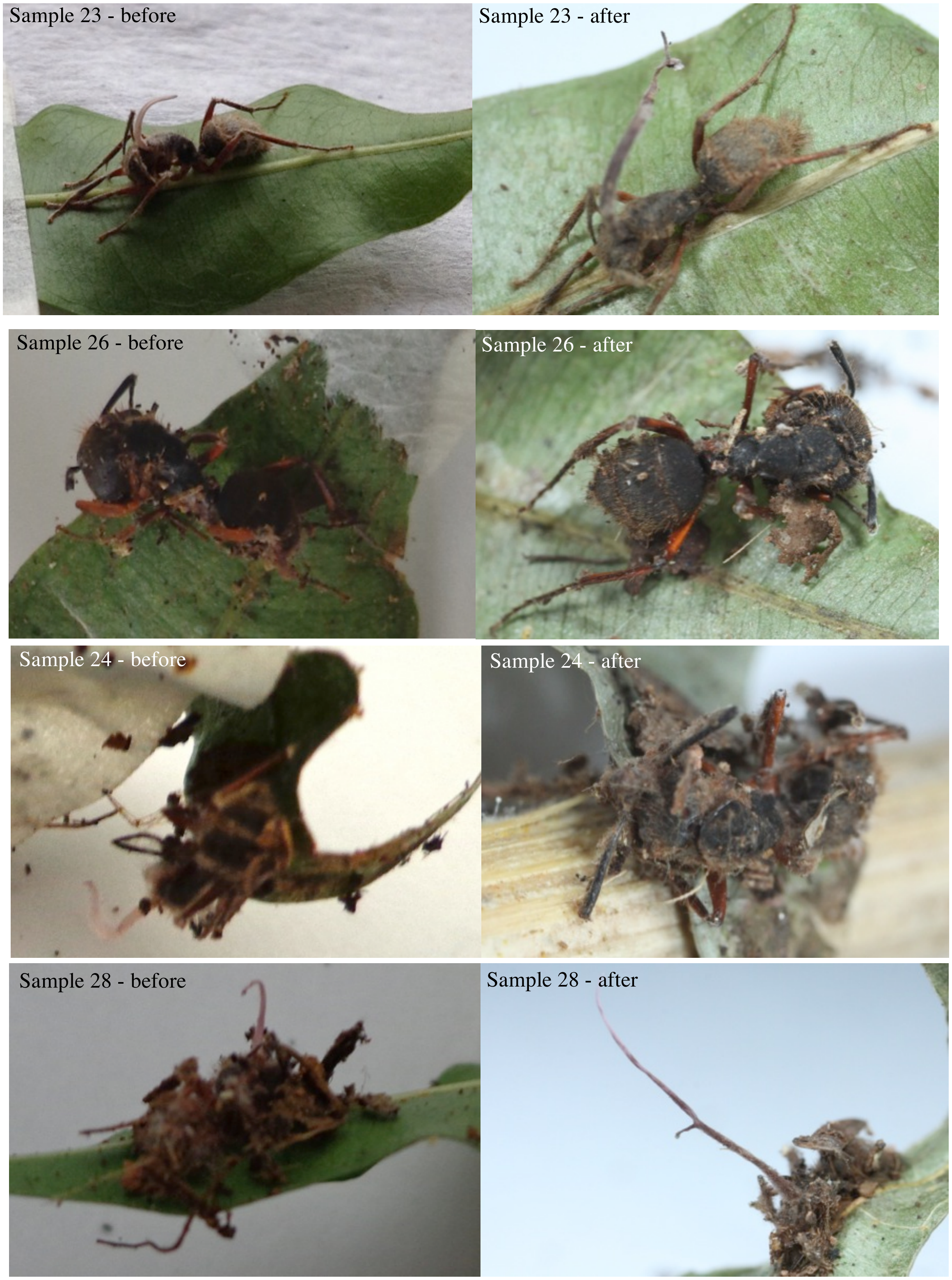
Sample 23 - before
Sample 23 - after
Sample 26 - before
Sample 26 - after
Sample 24 - before
Sample 24 - after
Sample 28 - before
Sample 28 - after
