# Supplementary material for: Long-Term Disease Dynamics for a Specialized Parasite of Ant Societies: A Field Study
Source: PLoS One. 2014 Aug 18;9(8):e103516. doi: 10.1371/journal.pone.0103516 (PMC4136743; doi:10.1371/journal.pone.0103516)
Supplement: Table S1 — Relative position (coordinates) of the 386 mapped infected ants found on the surrounding area of the four studied colonies across 20 months of field work. The data was collected in Atlantic rainforest, southeastern Brazil. The fungal parasite species is Ophiocordyceps camponoti-rufipedis that attacks the ant host Camponotus rufipes. The ant colonies were located in the center of the studied area and their coordinates are (x = 500, y = 500, z = 0). Months 1–6 correspond to December 2010 to May 2011. Month 14 corresponds to January 2012. (PDF) [file pone.0103516.s003.pdf]

| ant | x | y | z | month |
|---|---|---|---|---|
| 1 | 189 | 9 | 26 | 1 |
| 2 | 61 | 206 | 108 | 1 |
| 3 | 135 | 61 | 17 | 1 |
| 4 | 135 | 240 | 29 | 1 |
| 5 | 61 | 664 | 73 | 1 |
| 6 | 21 | 843 | 22 | 1 |
| 7 | 73 | 917 | 67 | 1 |
| 8 | 218 | 867 | 13 | 1 |
| 9 | 200 | 887 | 29 | 1 |
| 10 | 346 | 831 | 13 | 1 |
| 11 | 331 | 610 | 64 | 1 |
| 12 | 259 | 496 | 54 | 1 |
| 13 | 224 | 387 | 104 | 1 |
| 14 | 253 | 352 | 125 | 1 |
| 15 | 241 | 371 | 29 | 1 |
| 16 | 241 | 372 | 15 | 1 |
| 17 | 222 | 156 | 3 | 1 |
| 18 | 396 | 156 | 15 | 1 |
| 19 | 258 | 59 | 78 | 1 |
| 20 | 434 | 157 | 15 | 1 |
| 21 | 590 | 173 | 24 | 1 |
| 22 | 536 | 286 | 97 | 1 |
| 23 | 508 | 375 | 92 | 1 |
| 24 | 558 | 500 | 104 | 1 |
| 25 | 430 | 580 | 17 | 1 |
| 26 | 600 | 841 | 94 | 1 |
| 27 | 772 | 940 | 10 | 1 |
| 28 | 787 | 643 | 29 | 1 |
| 29 | 741 | 731 | 122 | 1 |
| 30 | 710 | 604 | 19 | 1 |
| 31 | 600 | 723 | 110 | 1 |
| 32 | 800 | 731 | 61 | 1 |
| 33 | 662 | 402 | 108 | 1 |
| 34 | 660 | 275 | 148 | 1 |
| 35 | 795 | 278 | 19 | 1 |
| 36 | 609 | 69 | 55 | 1 |
| 37 | 837 | 156 | 16 | 1 |
| 38 | 846 | 22 | 66 | 1 |
| 39 | 944 | 135 | 11 | 1 |
| 40 | 918 | 200 | 115 | 1 |
| 41 | 840 | 377 | 29 | 1 |
| 42 | 872 | 744 | 30 | 1 |
| 43 | 172 | 140 | 32 | 1 |
| 44 | 152 | 104 | 14 | 1 |
| 45 | 117 | 500 | 93 | 1 |
| 46 | 140 | 662 | 15 | 1 |
| 47 | 177 | 933 | 155 | 1 |

| | | | | |
|---|---|---|---|---|
| 48 | 41 | 906 | 112 | 1 |
| 49 | 334 | 200 | 23 | 1 |
| 50 | 288 | 28 | 6 | 1 |
| 51 | 394 | 154 | 128 | 1 |
| 52 | 348 | 393 | 51 | 1 |
| 53 | 305 | 452 | 13 | 1 |
| 54 | 318 | 550 | 100 | 1 |
| 55 | 222 | 691 | 16 | 1 |
| 56 | 508 | 986 | 113 | 1 |
| 57 | 542 | 809 | 38 | 1 |
| 58 | 528 | 826 | 23 | 1 |
| 59 | 502 | 724 | 135 | 1 |
| 60 | 547 | 551 | 16 | 1 |
| 61 | 547 | 546 | 20 | 1 |
| 62 | 400 | 572 | 26 | 1 |
| 63 | 432 | 447 | 56 | 1 |
| 64 | 502 | 253 | 131 | 1 |
| 65 | 461 | 215 | 27 | 1 |
| 66 | 430 | 186 | 16 | 1 |
| 67 | 489 | 40 | 22 | 1 |
| 68 | 632 | 45 | 81 | 1 |
| 69 | 607 | 19 | 58 | 1 |
| 70 | 589 | 21 | 82 | 1 |
| 71 | 776 | 237 | 100 | 1 |
| 72 | 774 | 283 | 142 | 1 |
| 73 | 713 | 648 | 29 | 1 |
| 74 | 662 | 495 | 38 | 1 |
| 75 | 759 | 905 | 30 | 1 |
| 76 | 980 | 850 | 34 | 1 |
| 77 | 983 | 782 | 14 | 1 |
| 78 | 1000 | 588 | 132 | 1 |
| 79 | 912 | 322 | 11 | 1 |
| 80 | 880 | 143 | 38 | 1 |
| 81 | 859 | 147 | 27 | 1 |
| 82 | 168 | 64 | 31 | 1 |
| 83 | 152 | 55 | 34 | 1 |
| 84 | 154 | 122 | 19 | 1 |
| 85 | 190 | 187 | 21 | 1 |
| 86 | 71 | 243 | 50 | 1 |
| 87 | 93 | 375 | 32 | 1 |
| 88 | 75 | 409 | 18 | 1 |
| 89 | 115 | 430 | 116 | 1 |
| 90 | 12 | 499 | 52 | 1 |
| 91 | 160 | 508 | 18 | 1 |
| 92 | 0 | 655 | 117 | 1 |
| 93 | 200 | 683 | 20 | 1 |
| 94 | 200 | 680 | 12 | 1 |
| 95 | 295 | 870 | 110 | 1 |

| | | | | |
|---|---|---|---|---|
| 96 | 360 | 973 | 40 | 1 |
| 97 | 263 | 663 | 33 | 1 |
| 98 | 317 | 682 | 23 | 1 |
| 99 | 311 | 682 | 36 | 1 |
| 100 | 419 | 460 | 12 | 1 |
| 101 | 560 | 520 | 69 | 1 |
| 102 | 446 | 902 | 21 | 1 |
| 103 | 793 | 870 | 35 | 1 |
| 104 | 739 | 600 | 99 | 1 |
| 105 | 757 | 525 | 19 | 1 |
| 106 | 726 | 555 | 102 | 1 |
| 107 | 687 | 425 | 40 | 1 |
| 108 | 632 | 413 | 41 | 1 |
| 109 | 629 | 216 | 99 | 1 |
| 110 | 697 | 337 | 38 | 1 |
| 111 | 614 | 59 | 58 | 1 |
| 112 | 692 | 31 | 79 | 1 |
| 113 | 978 | 188 | 104 | 1 |
| 114 | 993 | 170 | 69 | 1 |
| 115 | 994 | 658 | 29 | 1 |
| 116 | 987 | 906 | 16 | 1 |
| 117 | 190 | 22 | 119 | 1 |
| 118 | 174 | 104 | 143 | 1 |
| 119 | 105 | 137 | 115 | 1 |
| 120 | 200 | 924 | 123 | 1 |
| 121 | 372 | 0 | 91 | 1 |
| 122 | 327 | 113 | 80 | 1 |
| 123 | 258 | 84 | 41 | 1 |
| 124 | 293 | 86 | 28 | 1 |
| 125 | 362 | 215 | 52 | 1 |
| 126 | 268 | 283 | 46 | 1 |
| 127 | 244 | 263 | 31 | 1 |
| 128 | 238 | 263 | 19 | 1 |
| 129 | 366 | 518 | 54 | 1 |
| 130 | 200 | 411 | 122 | 1 |
| 131 | 266 | 553 | 24 | 1 |
| 132 | 297 | 858 | 102 | 1 |
| 133 | 237 | 889 | 18 | 1 |
| 134 | 268 | 981 | 29 | 1 |
| 135 | 433 | 990 | 75 | 1 |
| 136 | 507 | 610 | 90 | 1 |
| 137 | 468 | 490 | 36 | 1 |
| 138 | 417 | 200 | 86 | 1 |
| 139 | 564 | 94 | 98 | 1 |
| 140 | 683 | 185 | 110 | 1 |
| 141 | 701 | 224 | 98 | 1 |
| 142 | 766 | 298 | 123 | 1 |
| 143 | 628 | 800 | 69 | 1 |

| | | | | |
|---|---|---|---|---|
| 144 | 659 | 895 | 49 | 1 |
| 145 | 864 | 335 | 46 | 1 |
| 146 | 964 | 68 | 144 | 1 |
| 147 | 165 | 886 | 45 | 2 |
| 148 | 161 | 879 | 19 | 2 |
| 149 | 173 | 909 | 131 | 2 |
| 150 | 152 | 908 | 101 | 2 |
| 151 | 248 | 892 | 17 | 2 |
| 152 | 377 | 737 | 33 | 2 |
| 153 | 316 | 629 | 30 | 2 |
| 154 | 380 | 252 | 27 | 2 |
| 155 | 389 | 28 | 86 | 2 |
| 156 | 460 | 419 | 31 | 2 |
| 157 | 435 | 771 | 31 | 2 |
| 158 | 511 | 764 | 23 | 2 |
| 159 | 612 | 724 | 22 | 2 |
| 160 | 617 | 721 | 20 | 2 |
| 161 | 631 | 78 | 60 | 2 |
| 162 | 996 | 21 | 49 | 2 |
| 163 | 810 | 326 | 91 | 2 |
| 164 | 906 | 524 | 16 | 2 |
| 165 | 902 | 519 | 15 | 2 |
| 166 | 992 | 562 | 11 | 2 |
| 167 | 933 | 546 | 74 | 2 |
| 168 | 165 | 886 | 45 | 2 |
| 169 | 161 | 879 | 19 | 2 |
| 170 | 173 | 909 | 131 | 2 |
| 171 | 152 | 908 | 101 | 2 |
| 172 | 248 | 892 | 17 | 2 |
| 173 | 377 | 737 | 33 | 2 |
| 174 | 316 | 629 | 30 | 2 |
| 175 | 380 | 252 | 27 | 2 |
| 176 | 389 | 28 | 86 | 2 |
| 177 | 460 | 419 | 31 | 2 |
| 178 | 435 | 771 | 31 | 2 |
| 179 | 511 | 764 | 23 | 2 |
| 180 | 612 | 724 | 22 | 2 |
| 181 | 617 | 721 | 20 | 2 |
| 182 | 631 | 78 | 60 | 2 |
| 183 | 996 | 21 | 49 | 2 |
| 184 | 810 | 326 | 91 | 2 |
| 185 | 906 | 524 | 16 | 2 |
| 186 | 902 | 519 | 15 | 2 |
| 187 | 992 | 562 | 11 | 2 |
| 188 | 933 | 546 | 74 | 2 |
| 189 | 156 | 79 | 33 | 2 |
| 190 | 189 | 785 | 14 | 2 |
| 191 | 142 | 922 | 22 | 2 |

| | | | | |
|---|---|---|---|---|
| 192 | 135 | 830 | 41 | 2 |
| 193 | 330 | 464 | 23 | 2 |
| 194 | 222 | 583 | 17 | 2 |
| 195 | 261 | 382 | 62 | 2 |
| 196 | 243 | 260 | 31 | 2 |
| 197 | 258 | 290 | 10 | 2 |
| 198 | 420 | 351 | 22 | 2 |
| 199 | 579 | 448 | 27 | 2 |
| 200 | 428 | 996 | 102 | 2 |
| 201 | 653 | 390 | 51 | 2 |
| 202 | 600 | 381 | 48 | 2 |
| 203 | 702 | 128 | 56 | 2 |
| 204 | 817 | 6 | 16 | 2 |
| 205 | 826 | 785 | 94 | 2 |
| 206 | 873 | 817 | 90 | 2 |
| 207 | 909 | 856 | 38 | 2 |
| 208 | 917 | 848 | 36 | 2 |
| 209 | 97 | 182 | 70 | 2 |
| 210 | 34 | 464 | 52 | 2 |
| 211 | 69 | 639 | 16 | 2 |
| 212 | 65 | 723 | 131 | 2 |
| 213 | 343 | 929 | 70 | 2 |
| 214 | 335 | 944 | 13 | 2 |
| 215 | 270 | 387 | 86 | 2 |
| 216 | 226 | 328 | 20 | 2 |
| 217 | 229 | 234 | 94 | 2 |
| 218 | 335 | 219 | 110 | 2 |
| 219 | 239 | 8 | 140 | 2 |
| 220 | 272 | 1 | 41 | 2 |
| 221 | 261 | 0 | 43 | 2 |
| 222 | 551 | 292 | 64 | 2 |
| 223 | 532 | 294 | 30 | 2 |
| 224 | 462 | 469 | 29 | 2 |
| 225 | 441 | 518 | 71 | 2 |
| 226 | 437 | 746 | 124 | 2 |
| 227 | 935 | 177 | 26 | 2 |
| 228 | 818 | 43 | 48 | 2 |
| 229 | 852 | 668 | 106 | 2 |
| 230 | 82 | 191 | 141 | 3 |
| 231 | 25 | 880 | 87 | 3 |
| 232 | 105 | 882 | 33 | 3 |
| 233 | 366 | 829 | 47 | 3 |
| 234 | 235 | 848 | 62 | 3 |
| 235 | 378 | 769 | 123 | 3 |
| 236 | 207 | 277 | 155 | 3 |
| 237 | 212 | 405 | 17 | 3 |
| 238 | 262 | 180 | 29 | 3 |
| 239 | 259 | 78 | 59 | 3 |

| | | | | |
|---|---|---|---|---|
| 240 | 434 | 97 | 59 | 3 |
| 241 | 821 | 400 | 19 | 3 |
| 242 | 903 | 737 | 73 | 3 |
| 243 | 857 | 624 | 13 | 3 |
| 244 | 923 | 866 | 21 | 3 |
| 245 | 200 | 77 | 106 | 3 |
| 246 | 387 | 159 | 147 | 3 |
| 247 | 960 | 372 | 73 | 3 |
| 248 | 974 | 479 | 6 | 3 |
| 249 | 421 | 24 | 54 | 3 |
| 250 | 609 | 924 | 140 | 3 |
| 251 | 913 | 74 | 69 | 3 |
| 252 | 25 | 960 | 67 | 3 |
| 253 | 747 | 676 | 118 | 3 |
| 254 | 89 | 276 | 16 | 4 |
| 255 | 637 | 698 | 91 | 4 |
| 256 | 925 | 830 | 41 | 4 |
| 257 | 850 | 435 | 75 | 4 |
| 258 | 968 | 291 | 135 | 4 |
| 259 | 800 | 153 | 29 | 4 |
| 260 | 915 | 112 | 24 | 4 |
| 261 | 802 | 282 | 22 | 4 |
| 262 | 84 | 139 | 45 | 4 |
| 263 | 466 | 200 | 134 | 4 |
| 264 | 517 | 313 | 65 | 4 |
| 265 | 806 | 296 | 96 | 4 |
| 266 | 76 | 63 | 29 | 5 |
| 267 | 116 | 574 | 36 | 5 |
| 268 | 113 | 660 | 157 | 5 |
| 269 | 153 | 980 | 57 | 5 |
| 270 | 13 | 843 | 45 | 5 |
| 271 | 79 | 951 | 34 | 5 |
| 272 | 239 | 629 | 106 | 5 |
| 273 | 304 | 703 | 87 | 5 |
| 274 | 297 | 564 | 149 | 5 |
| 275 | 223 | 400 | 64 | 5 |
| 276 | 256 | 0 | 57 | 5 |
| 277 | 534 | 57 | 26 | 5 |
| 278 | 514 | 277 | 94 | 5 |
| 279 | 543 | 300 | 144 | 5 |
| 280 | 530 | 257 | 76 | 5 |
| 281 | 429 | 856 | 35 | 5 |
| 282 | 421 | 732 | 126 | 5 |
| 283 | 390 | 720 | 71 | 5 |
| 284 | 686 | 974 | 108 | 5 |
| 285 | 857 | 105 | 43 | 5 |
| 286 | 828 | 430 | 56 | 5 |
| 287 | 27 | 400 | 34 | 5 |

| | | | | |
|---|---|---|---|---|
| 288 | 181 | 683 | 111 | 5 |
| 289 | 226 | 849 | 136 | 5 |
| 290 | 271 | 414 | 35 | 5 |
| 291 | 539 | 734 | 25 | 5 |
| 292 | 575 | 658 | 101 | 5 |
| 293 | 717 | 298 | 135 | 5 |
| 294 | 800 | 345 | 79 | 5 |
| 295 | 115 | 67 | 21 | 5 |
| 296 | 383 | 873 | 23 | 5 |
| 297 | 445 | 72 | 21 | 5 |
| 298 | 655 | 97 | 120 | 5 |
| 299 | 91 | 849 | 58 | 5 |
| 300 | 720 | 891 | 67 | 5 |
| 301 | 46 | 23 | 75 | 5 |
| 302 | 107 | 394 | 146 | 5 |
| 303 | 117 | 454 | 30 | 5 |
| 304 | 21 | 910 | 13 | 5 |
| 305 | 258 | 940 | 63 | 5 |
| 306 | 400 | 348 | 130 | 5 |
| 307 | 876 | 579 | 92 | 5 |
| 308 | 69 | 138 | 108 | 6 |
| 309 | 78 | 600 | 88 | 6 |
| 310 | 451 | 284 | 80 | 6 |
| 311 | 450 | 635 | 82 | 6 |
| 312 | 440 | 626 | 83 | 6 |
| 313 | 609 | 690 | 94 | 6 |
| 314 | 676 | 645 | 44 | 6 |
| 315 | 714 | 836 | 19 | 6 |
| 316 | 664 | 881 | 88 | 6 |
| 317 | 758 | 903 | 51 | 6 |
| 318 | 781 | 957 | 90 | 6 |
| 319 | 678 | 800 | 94 | 6 |
| 320 | 815 | 400 | 67 | 6 |
| 321 | 175 | 166 | 130 | 6 |
| 322 | 400 | 516 | 78 | 6 |
| 323 | 467 | 254 | 11 | 6 |
| 324 | 521 | 450 | 37 | 6 |
| 325 | 724 | 870 | 20 | 6 |
| 326 | 682 | 832 | 48 | 6 |
| 327 | 809 | 660 | 99 | 6 |
| 328 | 313 | 695 | 58 | 6 |
| 329 | 282 | 696 | 55 | 6 |
| 330 | 278 | 37 | 21 | 6 |
| 331 | 445 | 903 | 14 | 6 |
| 332 | 655 | 878 | 43 | 6 |
| 333 | 823 | 586 | 27 | 6 |
| 334 | 132 | 506 | 14 | 6 |
| 335 | 120 | 626 | 16 | 6 |

| | | | | |
|---|---|---|---|---|
| 336 | 235 | 1000 | 47 | 6 |
| 337 | 228 | 523 | 93 | 6 |
| 338 | 187 | 400 | 72 | 6 |
| 339 | 182 | 410 | 58 | 6 |
| 340 | 435 | 174 | 102 | 6 |
| 341 | 418 | 583 | 81 | 6 |
| 342 | 488 | 552 | 112 | 6 |
| 343 | 427 | 986 | 88 | 6 |
| 344 | 763 | 911 | 122 | 6 |
| 345 | 780 | 267 | 87 | 6 |
| 346 | 845 | 149 | 35 | 6 |
| 347 | 867 | 400 | 31 | 6 |
| 348 | 85 | 917 | 89 | 14 |
| 349 | 676 | 703 | 79 | 14 |
| 350 | 283 | 431 | 37 | 14 |
| 351 | 238 | 154 | 36 | 14 |
| 352 | 627 | 124 | 162 | 14 |
| 353 | 784 | 168 | 53 | 14 |
| 354 | 845 | 86 | 19 | 14 |
| 355 | 884 | 353 | 18 | 14 |
| 356 | 851 | 290 | 48 | 14 |
| 357 | 852 | 283 | 33 | 14 |
| 358 | 871 | 394 | 24 | 14 |
| 359 | 800 | 411 | 14 | 14 |
| 360 | 881 | 460 | 22 | 14 |
| 361 | 909 | 857 | 41 | 14 |
| 362 | 154 | 891 | 131 | 14 |
| 363 | 284 | 978 | 53 | 14 |
| 364 | 373 | 721 | 121 | 14 |
| 365 | 460 | 989 | 68 | 14 |
| 366 | 387 | 600 | 78 | 14 |
| 367 | 251 | 544 | 127 | 14 |
| 368 | 209 | 182 | 37 | 14 |
| 369 | 545 | 179 | 55 | 14 |
| 370 | 509 | 242 | 104 | 14 |
| 371 | 470 | 757 | 45 | 14 |
| 372 | 417 | 625 | 21 | 14 |
| 373 | 701 | 852 | 23 | 14 |
| 374 | 695 | 591 | 8 | 14 |
| 375 | 612 | 416 | 18 | 14 |
| 376 | 150 | 898 | 34 | 14 |
| 377 | 226 | 421 | 80 | 14 |
| 378 | 516 | 731 | 34 | 14 |
| 379 | 503 | 878 | 25 | 14 |
| 380 | 672 | 989 | 49 | 14 |
| 381 | 743 | 108 | 46 | 14 |
| 382 | 274 | 529 | 12 | 14 |
| 383 | 282 | 552 | 25 | 14 |

| | | | | |
|---|---|---|---|---|
| 384 | 390 | 64 | 49 | 14 |
| 385 | 353 | 99 | 82 | 14 |
| 386 | 963 | 114 | 99 | 14 |
