# Supplementary material for: Long-Term Disease Dynamics for a Specialized Parasite of Ant Societies: A Field Study
Source: PLoS One. 2014 Aug 18;9(8):e103516. doi: 10.1371/journal.pone.0103516 (PMC4136743; doi:10.1371/journal.pone.0103516)
Supplement: Table S2 — Overview of co-evolved parasites in ant societies. Transmission can be between ants (direct) or also include another host (indirect). The final environment, where the sexual reproduction of the parasite occurs, can be in the environment surrounding the nest (Outside the nest), within the colony (Inside the nest) or final host (Vertebrate host). The effect of parasitism is often death of the infected, either directly attributable to the parasite (Direct death), or indirectly via a behavioral change that leads to the host being eaten by the final host (Predation) or jumping in water, to allow the parasite to enter water for mating (Drowning). Additional details of each group in Schmid-Hempel (1998) [6]. (DOCX) [file pone.0103516.s004.docx]

| **Overview of co-evolved parasites in ant societies.** | | | |
| --- | --- | --- | --- |
| **Parasite taxa** | **Transmission** | **Final Environment** | **Host consequence** |
| Virus | Direct | Unknown | Unknown |
| Bacteria | Direct | Unknown | Unknown |
| Fungi | Direct | Outside the nest | Direct death |
| Protozooa | Direct | Unknown | Unknown |
| Animal |  |  |  |
| Cestodes | Indirect | Vertebrate host | Predation |
| Trematodes | Indirect | Vertebrate host | Predation |
| Nematodes | Direct/Indirect | Water/ Vertebrate host | Drowning/Predation |
| Phorids | Direct | Outside the nest | Direct death |
| Strepsipterans | Direct | Outside the nest | Morbidity |

**Table S1:** **Overview of co-evolved parasites in ant societies.** Transmission can be between ants (direct) or also include another host (indirect). The final environment, where the sexual reproduction of the parasite occurs, can be in the environment surrounding the nest (Outside the nest), within the colony (Inside the nest) or final host (Vertebrate host). The effect of parasitism is often death of the infected, either directly attributable to the parasite (Direct death), or indirectly via a behavioral change that leads to the host being eaten by the final host (Predation) or jumping in water, to allow the parasite to enter water for mating (Drowning). Additional details of each group in Schmid-Hempel (1998) [6].
